# Multiscale Construction of Ag-Embedded PDMS Slippery Coatings on Titanium Alloy for Synergistic Antifouling Performance

**DOI:** 10.3390/ma18133090

**Published:** 2025-06-30

**Authors:** Yuyang Zhou, Yun Li, Hao Liu, Chi Ma, Jing Sun, Xin Liu

**Affiliations:** State Key Laboratory of High-Performance Precision Manufacturing, Dalian University of Technology, Dalian 116024, China; zyuyang12@163.com (Y.Z.); hebly@mail.dlut.edu.cn (Y.L.); 15141623006@163.com (H.L.); 15241796821@163.com (C.M.); sunjing@dlut.edu.cn (J.S.)

**Keywords:** slippery surface, PDMS, synergistic antifouling, laser etching

## Abstract

Low-surface-energy and wettability-based antifouling coatings have garnered increasing attention in marine applications owing to their environmentally friendly characteristics. However, their limited functionality often results in suboptimal long-term antifouling performance, particularly under dynamic marine conditions. To address these limitations, a polydimethylsiloxane (PDMS)-based slippery (PSL) coating was fabricated on TC4 titanium alloy by integrating surface silanization via (3-Aminopropyl)triethoxysilane (APTES), antimicrobial Ag-TiO_2_ nanoparticles, laser-induced hierarchical microtextures, and silicone oil infusion. The resulting PSL coating exhibited excellent oil retention and stable interfacial slipperiness even after thermal aging. Compared with bare TC4, low-surface-energy Ag-containing coatings, Ag-containing superhydrophobic coatings, and conventional slippery liquid-infused porous surfaces (SLIPS), the PSL coating demonstrated markedly superior resistance to protein adsorption, bacterial attachment, and diatom settlement, indicating an enhanced synergistic antifouling effect. Furthermore, it significantly reduced the diatom concentration in the surrounding medium without complete eradication, underscoring its eco-friendly and non-disruptive antifouling mechanism. This study offers a scalable, durable, and environmentally benign antifouling strategy for marine surface protection.

## 1. Introduction

Marine biofouling remains a significant challenge for submerged surfaces [1,2,3,4], particularly for metallic substrates such as TC4 titanium alloy. Owing to its excellent corrosion resistance and mechanical properties, TC4 is extensively used in marine structures, sensors, and biomedical implants [5]. However, long-term immersion in seawater inevitably leads to the accumulation of organic matter, microorganisms, and macrofoulers, which compromise surface functionality—referring to key properties such as wettability, surface energy, and microstructure—thereby increasing hydrodynamic drag and reducing equipment safety [1,6,7,8,9]. Among the current antifouling approaches, coating-based strategies are widely adopted due to their ease of application and cost-effectiveness. In the mid-20th century, self-polishing antifouling paints containing tributyltin (TBT) were commonly employed for their broad-spectrum efficacy [10]. Nevertheless, subsequent studies revealed that TBT posed severe ecological hazards, not only affecting target fouling organisms but also disrupting non-target marine ecosystems and exhibiting strong bioaccumulation [11,12]. As a result, the International Maritime Organization banned the use of TBT-based coatings in 2008, triggering a global demand for low-toxic or non-toxic, environmentally friendly alternatives [13,14].

In recent years, wettability-based antifouling methods have attracted increasing attention for their eco-friendly nature [15,16,17]. PDMS, in particular, possesses a low surface energy (20–26 mN·m^−1^) [18,19], falling within the Baier curve region that discourages fouling adhesion [20,21]. Compared with fluorinated polymers, PDMS exhibits slightly higher surface energy but benefits from a much lower elastic modulus, allowing for easier detachment of fouling species. Furthermore, the high bond dissociation energy of its Si–O backbone (~460 kJ·mol^−1^) endows PDMS with excellent thermal stability and oxidation resistance, making it a popular material in protective coatings. However, PDMS coatings are known to rely on high-speed water flow for effective foulant removal and tend to be less effective under static or low-flow marine conditions, especially against mucus layers formed by bacteria and diatoms. These limitations have significantly shortened their practical service life.

To enhance fouling resistance, researchers have drawn inspiration from nature, mimicking surface architectures found in shark skin, lotus leaves, and rose petals to construct superhydrophobic surfaces [22,23,24,25,26]. For example, flexible superhydrophobic PDMS coatings have been developed by spraying techniques, showing promise in waterproofing and fouling resistance [27]. Tian et al. [28] have integrated hierarchical structures and functional polymers to fabricate a superhydrophobic antifouling composite silicone for self-cleaning and antimicrobial behavior. However, these surfaces often suffer from mechanical fragility and instability in dynamic water environments. Once the trapped air layer collapses under pressure or impact, the rough structures become exposed, providing ample anchoring points for biofoulers.

In this context, slippery liquid-infused porous surfaces (SLIPS), inspired by the Nepenthes pitcher plant, offer a promising alternative [29,30,31]. SLIPS create a dynamic liquid interface that exhibits low adhesion and minimal fouling [32,33]. Their lubricating layer allows for self-repair and foulant removal with minimal external force. For instance, Liang et al. [34] developed a stable slippery organogel coating on carbon steel that effectively inhibited microbiologically influenced corrosion by suppressing microbial adhesion. Zhou et al. [35] synthesized a PDMS-based triblock copolymer and constructed a slippery surface through self-assembly and oil infusion, which showed excellent repellency and resistance to bacterial attachment compared to both smooth cured PDMS and microstructured superhydrophobic surfaces. However, the long-term performance of SLIPS is often hindered by lubricant depletion and instability in marine settings.

To overcome these limitations, synergistic antifouling strategies combining multiple functionalities are gaining attention. Polymer-based coatings that integrate antimicrobial agents, structural microfeatures, and liquid lubricants offer a comprehensive defense against fouling. PDMS, due to its strong chemical compatibility with silicone oils, serves as an ideal base for constructing slippery interfaces. By incorporating nanoscale fillers such as Ag-TiO_2_, it is possible to introduce active antimicrobial properties alongside passive slipperiness.

In this study, a multifunctional PDMS-based slippery coating (PSL) on TC4 titanium alloy was designed. The system combines (1) a PDMS matrix modified with APTES and Ag-TiO_2_ nanoparticles for chemical bonding and antimicrobial action; (2) laser-etched hierarchical microstructures to enhance oil storage; and (3) silicone oil infusion to establish a stable lubricating layer. Each component was systematically optimized for structural compatibility, oil retention, and fouling resistance. Surface characterization techniques were employed to assess morphology, wettability, and oil storage capacity. The antifouling performance was evaluated using protein, bacterial, and diatom adhesion tests under static marine conditions.

The resulting PSL coatings exhibited low sliding angles, strong oil retention, and robust resistance against multiple foulants. Building upon our previous findings that synergistic slippery surfaces outperformed conventional SLIPS and bare metal substrates [36,37], this work further enhances antifouling efficacy by optimizing the parameters of the content of coupling agent and nanoparticles, as well as the parameters of laser etching. Compared to bare TC4, low-surface-energy Ag-containing coatings, Ag-containing superhydrophobic coatings, and conventional SLIPS, the PSL coatings demonstrated superior antifouling performance, highlighting the advantages of a slippery and synergistic antifouling strategy. Notably, diatom assays confirmed that while Ag-containing coatings reduced algal concentration in the surrounding solution compared to bare TC4, they did not completely eliminate them—demonstrating a mild, non-lethal effect that highlights the coating’s eco-friendly character. These findings support the potential of PSL coatings as a scalable, environmentally benign solution for long-term marine biofouling mitigation on titanium-based materials.

## 2. Materials and Methods

### 2.1. Materials

Titanium alloy plates (TC4, Ti-6Al-4V, 60 mm × 30 mm × 2 mm) were acquired from Dongguan Zhuoyang Precision Technology Co., Ltd. (Dongguan, China). (3-Aminopropyl)triethoxysilane (APTES, C_9_H_23_NO_3_Si) was provided by Yousuo Chemical Technology Co., Ltd. (Qingdao, China). Hydroxyl-terminated PDMS (HTSO, HO[(CH_3_)_2_SiO]_n_H) was provided by Jinan Xingfeilong Chemical Co., Ltd. (Jinan, China). Titanium dioxide with a silver content of 1% (TiO_2_-Ag) was obtained from Zhitai Nano Micro New Material Co., Ltd. (Hangzhou, China). Dimethyl silicone oil ((CH_3_SiO[(CH_3_)_2_SiO]_n_Si(CH_3_)_3_, viscosity ~100 cs) was provided by Dow Corning Co., Ltd. (Freeland, MI, USA). Fluorosilane and anhydrous ethanol were purchased from Aladdin Biochemical Technology Co. (Shanghai, China). All chemicals were used as received, without further purification.

### 2.2. Preparation of Coatings

PDMS-based hydrophobic coating (PH) was built on TC4 surfaces by HTSO, APTES, and TiO_2_-Ag particles. PDMS-based superhydrophobic coating (PSH) was then obtained through a CO_2_ laser etching with processing parameters optimized by varying laser power (4.2–6.6 W), scan speed (200–400 mm/s), and line spacing (150–350 μm). A PDMS-based slippery coating (PSL) was fabricated after infusion of silicone oil. Slippery liquid-infused porous surfaces (SLIPS), a traditional slippery surface, were prepared by laser etching, fluorosilane modification, and silicone oil infusion [37].

### 2.3. Characterization of Coatings

The static contact angle (CA) and sliding angle (SA) were measured by the stopped-drop method using an optical contact angle meter (SL200KS, KINO Scientific Instrument Inc., Boston, MA, USA), fitted with the Asha–Young–Laplace equation, and tested at a rate of 12 frames/s. Surface morphology was examined using a field emission scanning electron microscope (SEM, SUPRA-55, ZEISS, Oberkochen, Germany) at magnifications of 50× and 200×. Surface roughness was measured by a white light interferometer (WLI, Newview 9000, ZYGO Corporation, Middlefield, CT, USA) using a 0.5× zoom lens in coherence scanning interferometry (CSI) mode, with a scan length of 65 μm and acquisition time of 6 s. Chemical groups were analyzed using a Fourier transform infrared spectrometer (FTIR, ThermoFisher IS50, Waltham, MA, USA) equipped with a DTGS detector, a resolution of 0.09 cm^−1^, and a scan range of 4000–500 cm^−1^. Protein and diatom concentrations were determined by a UV-Vis spectrophotometer (N4S, INESA, Yidian (Group) Co., Shanghai, China), with a wavelength range of 200–800 nm and a scan step size of 0.5 nm.

All measurements of CA, SA, and surface roughness were repeated five times at different regions of each sample, and the mean values were reported to ensure reproducibility and minimize random error. For the protein and diatom adhesion tests, three replicate samples were prepared for each coating type, and three replicate tests were analyzed for each independent sample to ensure the reliability and statistical validity of the data.

## 3. Results and Discussion

**Figure 1:**
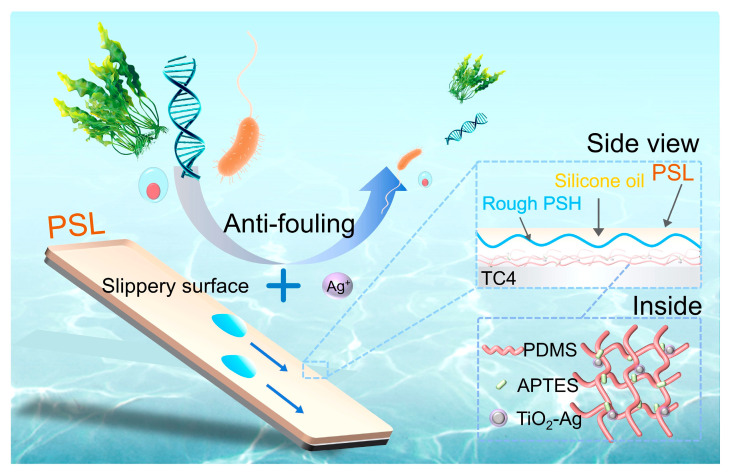
Schematic diagram of the synergistic antifouling mechanism on PSL coating.

To better illustrate the design rationale, Figure 1 presents a detailed schematic of the multifunctional a multifunctional PDMS-based slippery coating (PSL) structure and its synergistic antifouling mechanisms. The coating is composed of a PDMS-based matrix modified with APTES and uniformly embedded Ag-TiO_2_ nanoparticles, which provide long-lasting antimicrobial activity through controlled Ag^+^ release. Hierarchical microtextures are introduced via laser ablation to enhance oil retention and mechanical interlocking. The infused silicone oil forms a stable lubricating layer that minimizes interfacial adhesion and enables dynamic foulant removal. According to the fabrication principle of slippery surface, low surface energy, rough structure, and oil infusion were necessary. Therefore, the subsequent sections detail the construction and evaluation of each component layer and their collective performance.

### 3.1. Optimization of Synergistic Interface Composition and Microstructure

#### 3.1.1. Component Optimization for Low-Surface-Energy Coatings

To build a foundation for slippery surface construction, a PDMS-based smooth hydrophobic coating (PH) was formulated. Two key components—APTES and TiO_2_-Ag nanoparticles—were optimized due to their respective roles in adjusting crosslink density and introducing active antifouling sites.

**Figure 2:**
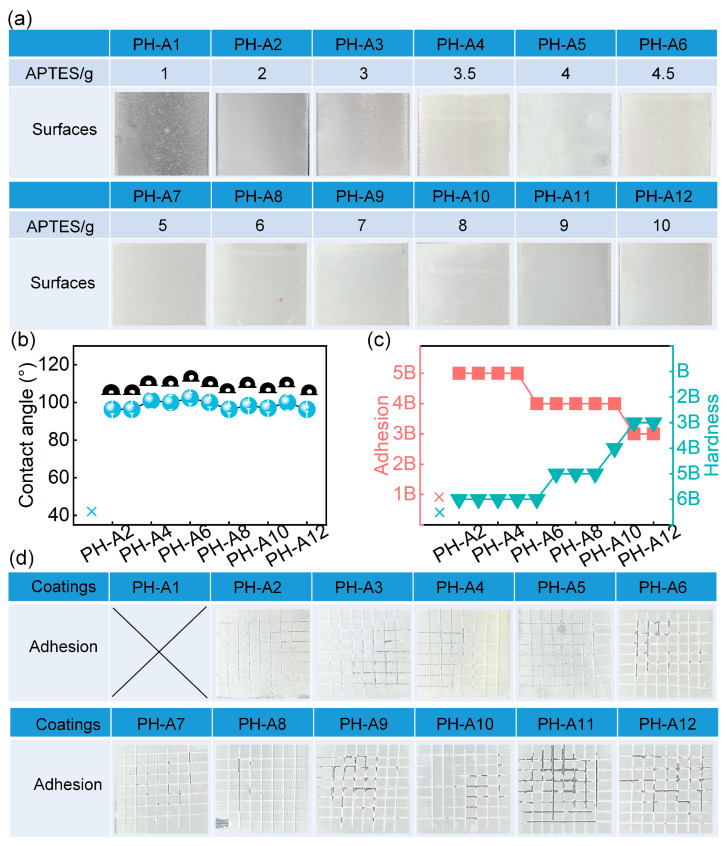
Effect of APTES content on surface wettability and mechanical properties. (**a**) Macroscopic appearance. (**b**) Static contact angle. (**c**) Adhesion and hardness strength of different coatings and (**d**) coating appearance after scratch adhesion testing.

APTES content was first varied to modulate the network structure of the PDMS matrix (10 g HTSO and 1.3 wt.% TiO_2_-Ag). As shown in Figure 2, an increase in APTES enhanced crosslinking density, thereby increasing hardness while reducing flexibility. This resulted in compromised film formation at low APTES contents (PH-A1 in Figure 2). Hydrophobicity remained above 90°, with a peak value of 100.5° on the PH-A7 surface, measured using a 5 μL water droplet. (Figure 2b). Adhesion strength (ASTM D3359 [38]) decreased while hardness (GB/T 6739-2022 [39]) increased with APTES dosage, confirming enhanced rigidity (Figure 2c). Scratch images (Figure 2d) further validated film integrity. Based on balanced wettability and mechanical performance, PH-A7 was selected.

**Figure 3:**
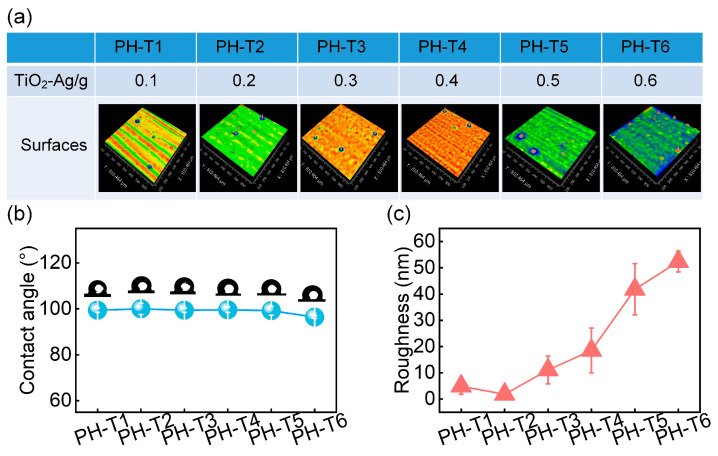
Effect of TiO_2_-Ag particle content on surface wettability and roughness. (**a**) 3D surface topography. (**b**) Contact angle and (**c**) surface roughness.

Building on the optimized APTES-modified PDMS matrix (5 g APTES and 10 g HTSO), TiO_2_-Ag nanoparticles were subsequently incorporated to impart active antifouling functionality. In addition to their anti-bacterial role, the presence of these nanoparticles also altered surface roughness and wettability due to their inherent surface properties. Therefore, contact angle and surface roughness were chosen to evaluate their influence. As shown in Figure 3, excessive particle loading slightly reduced hydrophobicity due to the moisture affinity of the nanoparticle surface (Figure 3b). Roughness first decreased, then increased with particle content (Figure 3c), which is attributed to an initial smoothing effect followed by the formation of protrusions due to nanoparticle aggregation (Figure 3a). The PH-T2 formulation (1.3 wt.%) achieved the optimal morphology and contact angle.

Thus, the optimized PH base coating was formulated using HTSO:APTES = 2:1 and TiO_2_-Ag = 1.3 wt.%, providing low surface energy, suitable roughness, and reliable mechanical integrity for subsequent structuring.

#### 3.1.2. Laser-Induced Microstructures for Superhydrophobicity Transition

To create the hierarchical roughness required for oil retention and slippery behavior, the optimized PH coating was further modified via laser texturing. Three laser processing parameters—laser power, scan speed, and scan line spacing—were systematically investigated. Each parameter affects energy deposition and therefore determines the resulting surface morphology and wettability.

**Figure 4:**
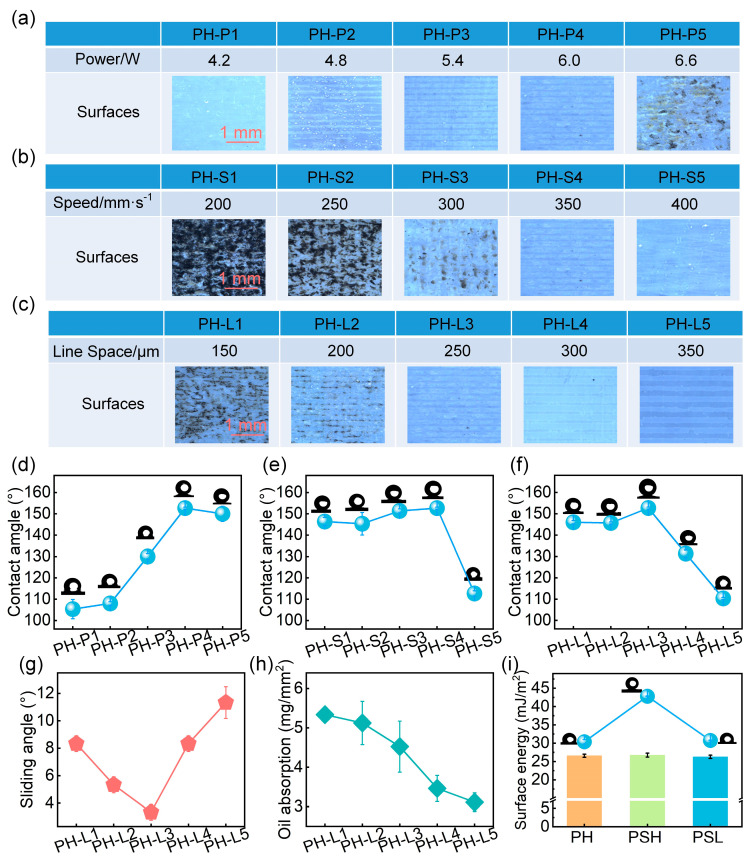
Effect of laser parameters on surface morphology, wettability, and structure for oil storage. Morphology of surfaces treated with varying (**a**) laser power, (**b**) scanning speed, and (**c**) scanning line space. Static contact angle on surfaces with different (**d**) laser power, (**e**) scanning speed, and (**f**) scanning line space. (**g**) Sliding angle of coatings under different laser spacing. (**h**) Oil absorption capacity under different laser spacing and (**i**) surface energy of coatings with different wettability.

Laser power primarily controls the extent of ablation. As shown in Figure 4a, low power resulted in insufficient etching, while excessive power caused surface damage due to overheating. Contact angle increased with power and reached a maximum of 153° at 6 W (Figure 4d), indicating optimal microstructure formation for superhydrophobicity.

Scan speed inversely affected energy input. At very low speed, overexposure led to surface burning and collapse, while excessive speed yielded weak texturing (Figure 4b). Contact angle decreased significantly when the scan speed was too high (Figure 4e), reflecting insufficient structure formation.

Scan line spacing determined the periodicity and uniformity of the laser-induced structures. Narrow spacing led to overlapping ablation and surface degradation, while wide spacing resulted in insufficient texture formation and reduced hydrophobicity (Figure 4c,f). Further analysis revealed that oil absorption capacity decreased progressively with increasing spacing, whereas the sliding angle exhibited a non-linear trend—initially decreasing and then increasing (Figure 4g,h). The surface textured with a 250 μm line spacing demonstrated optimal performance, combining a low sliding angle (3.4°) with high oil storage capacity.

Based on these results, the laser parameters of 6 W power, 350 mm/s scanning speed, and 250 μm line spacing were selected. The resulting PDMS-based superhydrophobic coating (PSH) exhibited uniform microstructures and excellent oil-retaining ability and was subsequently infused with silicone oil to form the final multifunctional PSL coating. Surface energy measurements confirmed that the PH, PSH, and PSL surfaces (25–27 mN/m) all fell within the Baier fouling-resistant range, fulfilling the criteria for constructing an effective slippery interface (Figure 4i).

### 3.2. Structural Characterization and Oil Storage Capability

**Figure 5:**
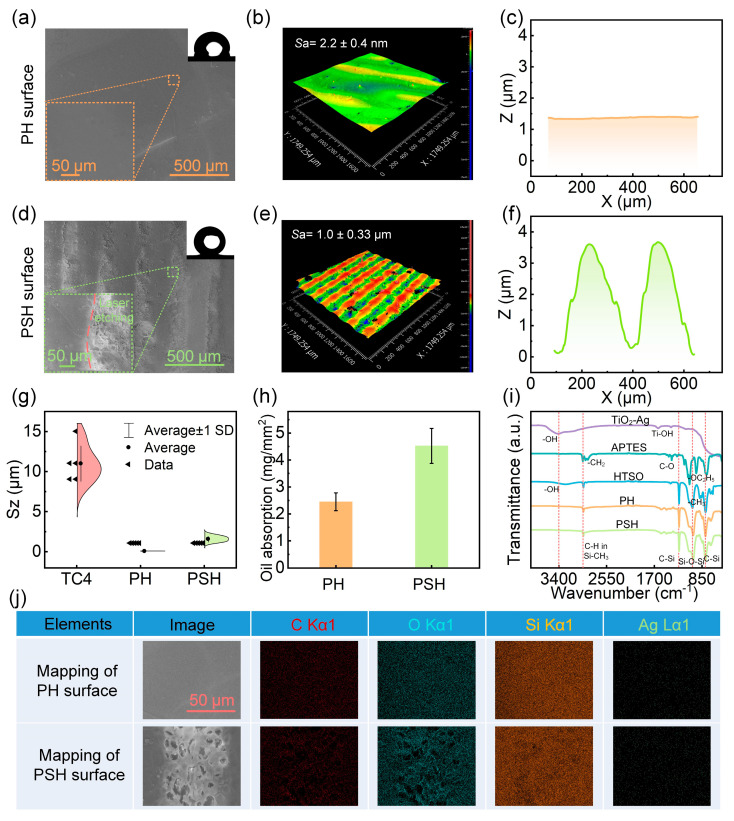
Characterization of the PH and PSH surfaces before and after laser structuring. (**a**) SEM image, (**b**) 3D surface topography, and (**c**) cross-sectional graphs of PH surface. (**d**) SEM image, (**e**) 3D surface topography and (**f**) cross-sectional graphs of PSH surface. (**g**) Maximum height difference (Sz) values of surface profiles. (**h**) Oil storage ability of PH and PSH coatings. (**i**) FTIR spectra and (**j**) elements distribution on PH and PSH surfaces.

Figure 5 characterizes the morphological and chemical features of the PH and PSH surfaces. The SEM image of the PH coating (Figure 5a) shows a flat and featureless surface. White-light interferometry reveals a surface roughness (*S*a) of only 2.2 nm (Figure 5b), and the corresponding cross-sectional profile (Figure 5c) confirms its smooth morphology. After laser treatment, the PSH surface exhibits an array of well-defined microstructures. As shown in Figure 5d, the ablated regions are rough with distinct microtextures, while the non-ablated areas remain smooth. The 3D surface topography (Figure 5e) and side profile (Figure 5f) clearly show the periodic concave–convex patterns introduced by laser ablation, which are favorable for oil storage.

The surface height distributions (Figure 5g) indicate that the raw TC4 substrate has an uneven and irregular height profile. The PH coating effectively smooths out the native surface defects of TC4. In contrast, the PSH surface, while exhibiting increased roughness, shows more uniform height variations, suggesting the formation of regular microtextures. This structural regularity contributes to enhanced oil retention, as supported by the results in Figure 5h. Compared to PH, the PSH coating exhibits nearly twice the oil storage capacity. This is attributed to the combination of chemical compatibility between the PDMS-derived siloxane network and silicone oil, as well as the structural benefit of the microreservoirs formed via laser ablation.

FTIR spectra (Figure 5i) reveal that the PH coating is formed through condensation reactions between APTES and HTSO, encapsulating hydroxyl-rich Ag-TiO_2_ particles within the siloxane matrix. The persistent absorption peaks of Si–CH_3_ (2962 cm^−1^), Si–O–Si (1093 cm^−1^), and C–Si (1229 cm^−1^, 864 cm^−1^) after curing indicate that the surface retains its low-energy siloxane backbone [40,41,42,43]. The similarity of PSH’s FTIR profile to that of PH suggests that laser ablation does not significantly alter the chemical composition. Instead, it primarily contributes to physical structuring. Furthermore, elemental mapping (Figure 5j) shows a homogeneous distribution of key elements (C, O, Si, Ag) across both PH and PSH surfaces, confirming consistent dispersion of the functional components.

### 3.3. Slippery and Antifouling Properties of PSL Coating

**Figure 6:**
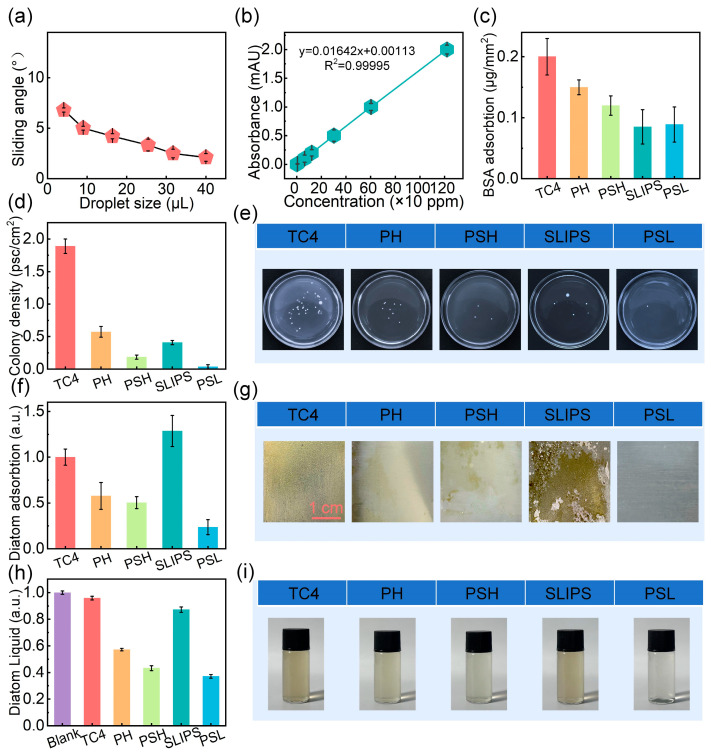
Slippery behavior and antifouling performance comparison among different surfaces. (**a**) Sliding angle of water droplets of different sizes on PSL surface. (**b**) Standard protein adsorption curve. (**c**) Anti-protein adhesion performance, (**d**,**e**) anti-bacterial attachment performance, and (**f**–**i**) anti-diatom performance of TC4, PH, PSH, SLIPS, and PSL surfaces.

The PSL coating, formed by infusing the structured PSH surface with silicone oil, demonstrates excellent slippery behavior. As shown in Figure 6a, water droplets of various sizes readily slide off the surface with minimal sliding angles, indicating stable lubricant retention and low interfacial friction—critical prerequisites for dynamic fouling resistance in marine environments.

To evaluate the antifouling performance, five surfaces were compared: (i) bare TC4, (ii) PH (Ag-containing low-surface-energy coating), (iii) PSH (Ag-containing superhydrophobic surface), (iv) traditional slippery liquid-infused porous surfaces (SLIPS), and (v) PSL (Ag-containing slippery surface). Notably, both PH and PSH incorporate Ag to achieve dual-mode antifouling through surface chemistry and biocidal effects, while PSL integrates Ag release with lubricant-induced interfacial mobility, achieving a synergistic dual-function antifouling mechanism. Marine biofouling typically proceeds in three major stages: (1) adsorption of dissolved organic molecules (e.g., proteins) forming a conditioning film, (2) attachment of microorganisms such as bacteria to initiate biofilm formation, and (3) colonization by larger organisms, including diatoms and macrofoulers. To mimic these processes, three representative foulants were selected in this study: bovine serum albumin (BSA), *S. aureus*, and marine diatoms.

In the initial conditioning film formation stage, protein fouling was assessed by immersing each sample in 50 mL of BSA solution for 12 h to reach adsorption–desorption equilibrium. The protein concentration was determined by UV–Vis spectroscopy, and the surface adsorption (*A*, μg/mm^2^) was calculated using Equation (1) [44]:

(1) $$A=\frac{V}{S}\times (C_{0}-C_{1})\times 100\%$$

where *V* is the volume of protein solution, *S* is the effective adsorption area of the sample, *C*_0_ is the protein concentration of the initial standard solution, and *C*_1_ is the protein concentration of the remaining solution after adsorption equilibrium is reached. According to the standard curve (Figure 6b), the results in Figure 6c indicate that both SLIPS and PSL coatings significantly suppress protein adsorption, in stark contrast to TC4, PH, and PSH. This demonstrates that lubricant-infused surfaces effectively minimize molecular adhesion, which highlights the effectiveness of the lubricant layer in resisting initial fouling.

During the second stage, biofilm formation was evaluated by culturing samples in *S. aureus* suspension for 24 h. As shown in Figure 6d,e, TC4 exhibited the highest bacterial density (~1.9 psc/cm^2^). PH and SLIPS coatings moderately reduced adhesion through either Ag^+^ release or surface slipperiness, while PSH further suppressed colonization due to its superhydrophobic microstructure (~0.185 psc/cm^2^). Notably, PSL achieved the lowest bacterial attachment (~0.037 psc/cm^2^), highlighting the synergistic effect of lubricant-induced motility barriers and sustained Ag^+^ release. These findings confirm that PSL surpasses both the PH-type low-surface-energy/Ag^+^ coating and the PSH-type superhydrophobic/Ag^+^ coating in resisting microbial contamination, as well as traditional SLIPS with single-function antifouling.

In the third stage of fouling, marine diatom adhesion was investigated. Figure 6f–i show that SLIPS, despite its slipperiness, exhibits substantial diatom settlement—likely due to the absence of biocidal agents and loss of lubricant layer. PH and PSH coatings moderately reduce adhesion, but PSL achieves the lowest diatom density. This result confirms that the PSL coating’s dual-mode antifouling—comprising physical mobility and chemical bactericidal effects—is highly effective against microfouling organisms. Additionally, the surrounding medium of PH, PSH, and PSL showed lower diatom concentrations than the control, indicating that Ag release plays a supplementary role in suppressing bioaccumulation. As depicted in Figure 6f,g, SLIPS showed significant diatom settlement, likely due to partial lubricant depletion and lack of biocidal agents. PH and PSH exhibited moderate suppression, attributable to Ag^+^-mediated biocidal effects. In contrast, PSL again exhibited the lowest diatom density, reflecting superior long-term resistance through dual-function antifouling—physically reducing adhesion via lubrication and chemically inhibiting growth via Ag^+^ diffusion. Furthermore, the surrounding medium of PH, PSH, and PSL showed noticeably lower planktonic diatom density (Figure 6h,i). This suggests that the coatings effectively inhibited diatom adhesion without completely killing the planktonic organisms. By employing a non-toxic, low-adhesion strategy, the coatings reduce biofouling pressure while minimizing ecological disruption, thus exhibiting a green and environmentally friendly antifouling mechanism.

Taken together, the PSL coating integrates low interfacial adhesion, dynamic lubricant flow, and chemical bactericidal action, outperforming traditional and dual-functional coatings. Its robust slippery surface and comprehensive antifouling mechanism offer promising potential for durable marine antifouling applications.

### 3.4. Thermal Stability of PSL Coating

**Figure 7:**
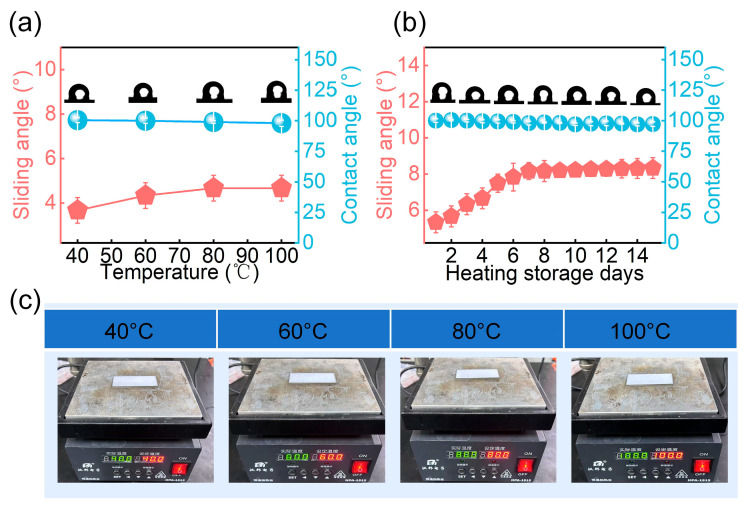
Thermal stability of the PSL coating under heating conditions. (**a**) Changes in contact angle and sliding angle after 30 min of heating from 40 °C to 100 °C. (**b**) Evolution of sliding angle and contact angle after 15 days of storage at 100 °C, and (**c**) digital image of heating process.

To assess durability, the thermal stability of the PSL coating was examined (Figure 7). After 30 min of heating from 40 °C to 100 °C, the contact angle remained nearly unchanged, while the sliding angle slightly increased but stayed below 10°, indicating stable lubricant retention (Figure 7a,c). Long-term heating at 100 °C for 15 days led to a minor decrease in contact angle, yet the sliding angle stabilized at ~8° after 7 days (Figure 7b). These results suggest that the PSL surface maintains its slippery behavior and wetting characteristics under thermal stress, supporting its potential for long-term antifouling use in elevated temperature environments.

## 4. Conclusions

A multifunctional slippery antifouling coating (PSL) was fabricated on TC4 by integrating APTES-modified PDMS, Ag-TiO_2_ nanoparticles, laser-induced textures, and silicone oil. The PSL surface exhibited low sliding angle (3.4°), strong oil retention, and low surface energy (26.1 mN/m). It outperformed bare TC4, PH surface (an Ag-containing low-surface-energy surface), PSH surface (an Ag-containing superhydrophobic surface), and conventional SLIPS, reducing protein, bacterial, and diatom adhesion by 56.7%, 98.1%, and 81.72%, respectively. After thermal aging at 100 °C for 15 days, the sliding angle remained stable (~8°), indicating good long-term durability. This study highlights a scalable, eco-friendly strategy for robust marine antifouling.

## Data Availability

The original contributions presented in this study are included in the article. Further inquiries can be directed to the corresponding author.

## References

[1] Wan F., Yan W., Feng C., Tong R., Zhang L. (2023). Preparation and Study of Antifouling and Fouling-Release Surface Materials From Copolymers with Anchoring Functional Groups. Materials.

[2] Wang C., Chen R., Liu W., Yu J., Liu Q., Liu J., Zhu J., Lin C., Li Y., Wang J. (2025). Electron-Withdrawing Effects for Tailoring Oxidative-Stress-Mediated Coating in Marine Antifouling. ACS Appl. Mater. Interfaces.

[3] Zhao W., Wu Z., Liu Y., Dai P., Hai G., Liu F., Shang Y., Cao Z., Yang W. (2023). Research Progress of Natural Products and their Derivatives in Marine Antifouling. Materials.

[4] Zhang S., Shen Y., Yan Y., Guo F., Shi W. (2025). Integrated CuO/G-C3N4 S-Scheme Heterojunction Self-Healing Coatings: A Synergistic Approach for Advanced Anti-Corrosion and Anti-Biofouling Performance. J. Mater. Sci. Technol..

[5] Wang X., Zhu S., Yang Z., Wang C., Wang N., Zhang Y., Yu F. (2022). Corrosion–Resistance Mechanism of TC4 Titanium Alloy Under Different Stress-Loading Conditions. Materials.

[6] Liu X., Sun J., Duan J., Sui K., Zhai X., Zhao X. (2024). AgNP Composite Silicone-Based Polymer Self-Healing Antifouling Coatings. Materials.

[7] Liu X., Zou L., Li B., Di Martino P., Rittschof D., Yang J., Maki J., Liu W., Gu J. (2024). Chemical Signaling in Biofilm-Mediated Biofouling. Nat. Chem. Biol..

[8] Wu X., Xiao M., Zhang J., Tan G., Pan Y., Lai Y., Chen Z. (2023). An Underwater Stable Superhydrophobic Surface for Robust Ultra-Long-Lasting Biofouling Resistance. Chem. Eng. J..

[9] Zhang P., Lin L., Zang D., Guo X., Liu M. (2016). Designing Bioinspired Anti-Biofouling Surfaces Based On a Superwettability Strategy. Small.

[10] Hong H., Lv J., Deng A., Tang Y., Liu Z. (2024). A Review of Experimental Assessment Processes of Material Resistance to Marine and Freshwater Biofouling. J. Environ. Manage..

[11] Arsenie L.V., Bangoura M.A., Ramonda M., Merindol R., Hespel L., Blanquer S., Azemar F., Lapinte V. (2025). Photo-Switchable Polyoxazoline Additive for Marine Fouling Release Silicone Coatings. Prog. Org. Coat..

[12] Sousa R.P.C.L., Figueira R.B., Costa S.P.G., Raposo M.M.M. (2020). Optical Fiber Sensors for Biocide Monitoring: Examples, Transduction Materials, and Prospects. ACS Sens..

[13] Gu Y., Yu L., Mou J., Wu D., Xu M., Zhou P., Ren Y. (2020). Research Strategies to Develop Environmentally Friendly Marine Antifouling Coatings. Mar. Drugs.

[14] Tian L., Yin Y., Jin H., Bing W., Jin E., Zhao J., Ren L. (2020). Novel Marine Antifouling Coatings Inspired by Corals. Mater. Today Chem..

[15] Liu Y., Gu H., Jia Y., Liu J., Zhang H., Wang R., Zhang B., Zhang H., Zhang Q. (2019). Design and Preparation of Biomimetic Polydimethylsiloxane (PDMS) Films with Superhydrophobic, Self-Healing and Drag Reduction Properties Via Replication of Shark Skin and SI-ATRP. Chem. Eng. J..

[16] Zhou Y., Li Y., Chen Y., Cao X., Zhang Y., Wang S., Liu J., Liu X. (2023). Environmentally Friendly Super-Slippery Concrete with Anti-Corrosion and Anti-Fouling Properties. Mater. Lett..

[17] Peta K., Bartkowiak T., Rybicki M., Galek P., Mendak M., Wieczorowski M., Brown C.A. (2024). Scale-Dependent Wetting Behavior of Bioinspired Lubricants On Electrical Discharge Machined Ti6Al4V Surfaces. Tribol. Int..

[18] He X., Wang T., Huang J., Chen J., Li J. (2020). Fabrication and Characterization of Superhydrophobic PDMS Composite Membranes for Efficient Ethanol Recovery Via Pervaporation. Sep. Purif. Technol..

[19] Ma R., Lu X., Zhang S., Ren K., Gu J., Liu C., Liu Z., Wang H. (2022). Constructing Discontinuous Silicon-Island Structure with Low Surface Energy Based On the Responsiveness of Hydrophilic Layers to Improve the Anti-Fouling Property of Membranes. J. Membr. Sci..

[20] Akhtar N., Bowen J., Asteriadou K., Robbins P.T., Zhang Z., Fryer P.J. (2010). Matching the Nano- To the Meso-Scale: Measuring Deposit–Surface Interactions with Atomic Force Microscopy and Micromanipulation. Food Bioprod. Process..

[21] Finlay J.A., Bennett S.M., Brewer L.H., Sokolova A., Clay G., Gunari N., Meyer A.E., Walker G.C., Wendt D.E., Callow M.E. (2010). Barnacle Settlement and the Adhesion of Protein and Diatom Microfouling to Xerogel Films with Varying Surface Energy and Water Wettability. Biofouling.

[22] Avrămescu R., Ghica M.V., Dinu-Pîrvu C., Prisada R., Popa L. (2018). Superhydrophobic Natural and Artificial Surfaces—A Structural Approach. Materials.

[23] González Lazo M., Katrantzis I., Dalle Vacche S., Karasu F., Leterrier Y. (2016). A Facile in Situ and UV Printing Process for Bioinspired Self-Cleaning Surfaces. Materials.

[24] Shao Y., Zhao J., Fan Y., Wan Z., Lu L., Zhang Z., Ming W., Ren L. (2020). Shape Memory Superhydrophobic Surface with Switchable Transition Between “Lotus Effect” to “Rose Petal Effect”. Chem. Eng. J..

[25] Xiang Y., Huang S., Lv P., Xue Y., Su Q., Duan H. (2018). Ultimate Stable Underwater Superhydrophobic State. Phys. Rev. Lett..

[26] Xu X., Shi L., Wang S. (2020). An Innovative Armour-Strategy for Robust Superhydrophobic Surfaces. Sci. China Chem..

[27] Xing L., Zhang Q., Yu J., Huang X., Gong X. (2024). Preparation and Applicability of a Flexible PDMS Superhydrophobic Layer. Mater. Today Commun..

[28] Tian D., Zhang K., Sun L., Rong Z., Zhang D., Liu L., Wu Y., Gao C., Kan Z., Liu Y. (2025). Renewable Superhydrophobic Antifouling Composite Silicone Based On Micro-Nano Structure. Compos. Sci. Technol..

[29] Li H., Yan M., Zhao W. (2022). Designing a MOF-Based Slippery Lubricant-Infused Porous Surface with Dual Functional Anti-Fouling Strategy. J. Colloid. Interface. Sci..

[30] Manna U., Raman N., Welsh M.A., Zayas Gonzalez Y.M., Blackwell H.E., Palecek S.P., Lynn D.M. (2016). Slippery Liquid-Infused Porous Surfaces that Prevent Microbial Surface Fouling and Kill Non-Adherent Pathogens in Surrounding Media: A Controlled Release Approach. Adv. Funct. Mater..

[31] Zhou Y., Chen G., Ma J., Li Y., Cao X., Xu Y., Song J., Liu X. (2022). Slippery Concrete for Sanitation. Prog. Org. Coat..

[32] Guo Y., Yan M., Zhao W. (2024). Cinnamaldehyde Grafted Porous Aerogel-Organ Gel Liquid Infused Surface for Achieving Difunctional Long-Term Dynamic Antifouling. J. Colloid. Interface. Sci..

[33] He J., Li J., Sun Y., Shen Y., Wei Q., Zhang D., Feng D., Wang P. (2025). Molecular Mechanism of Oil-Infused Silicone Preventing Mussel Biofouling. Research.

[34] Liang Y., Wang P., Zhang D. (2021). Designing a Highly Stable Slippery Organogel on Q235 Carbon Steel for Inhibiting Microbiologically Influenced Corrosion. ACS Appl. Bio. Mater..

[35] Zhou X., Lee Y.-Y., Chong K.S.L., He C. (2018). Superhydrophobic and Slippery Liquid-Infused Porous Surfaces Formed by the Self-Assembly of a Hybrid ABC Triblock Copolymer and their Antifouling Performance. J. Mat. Chem. B.

[36] Li Y., Zhou Y., Lin J., Liu H., Liu X. (2024). Antifouling Slippery Surface with Enhanced Stability for Marine Applications. Materials.

[37] Zhou Y., Cao X., Chen Y., Li Y., Ma J., Song J., Liu X. (2023). Blue-Ringed Octopus Inspired Slippery Coating with Physico-Chemical Synergistic Antifouling Properties. Chem. Eng. J..

[38] (2017). Standard Test Methods for Rating Adhesion by Tape Test.

[39] (2022). Paints and varnishes—Determination of film hardness by pencil test.

[40] Bai Y., Jiang X., He B., Zhu Y., Zhang Y. (2024). Polydimethylsiloxane Enabled Triple-Action Water-Resistant Coating with Desirable Relaxation Rate in Clear Aligner. J. Colloid. Interface. Sci..

[41] Li X., Zhang L., Yang Z., He Z., Wang P., Yan Y., Ran J. (2020). Hydrophobic Modified Activated Carbon Using PDMS for the Adsorption of VOCs in Humid Condition. Sep. Purif. Technol..

[42] Rodrigues A.P., Santos P.M.P., Veiga J.P., Casimiro M.H., Ferreira L.M. (2023). Electron Beam Irradiation On the Production of a Si- And Zr-Based Hybrid Material: A Study by FTIR and WDXRF. Materials.

[43] Tsuzuki T., Baassiri K., Mahmoudi Z., Perumal A.S., Rajendran K., Rubies G.M., Nicolau D.V. (2022). Hydrophobic Recovery of PDMS Surfaces in Contact with Hydrophilic Entities: Relevance to Biomedical Devices. Materials.

[44] Zhou Z., Wang H., Zhang X., Zhang Z., Yang L. (2025). Mechanistic Interaction Studies on Variform PCN-224 Nanoparticles with Transferrin and Lysozyme. J. Mol. Struct..

